# Anti-inflammatory and Anti-oxidative Effects of Phytohustil^®^ and Root Extract of *Althaea officinalis* L. on Macrophages *in vitro*

**DOI:** 10.3389/fphar.2020.00290

**Published:** 2020-03-17

**Authors:** Gabriel A. Bonaterra, Kevin Bronischewski, Pascal Hunold, Hans Schwarzbach, Ennio-U. Heinrich, Careen Fink, Heba Aziz-Kalbhenn, Jürgen Müller, Ralf Kinscherf

**Affiliations:** ^1^Department of Medical Cell Biology, Anatomy and Cell Biology, University of Marburg, Marburg, Germany; ^2^Bayer Consumer Health Division, Phytomedicines Supply and Development Center, Steigerwald Arzneimittelwerk GmbH, Darmstadt, Germany

**Keywords:** *Althaea officinalis* L., anti-inflammatory, anti-oxidative, macrophage, marshmallow, migration, Phytohustil^®^, ROS

## Abstract

**Introduction:**

The medicinal plant marshmallow *Althaea officinalis* L. (*A. officinalis*), is used for the treatment of cough since centuries. Application of medicinal extracts of marshmallow roots shows immediate effects like a protective film on the inflamed mucosa. Because the soothing layer reduce irritation of the mucous system, a faster regeneration is supported by defense mechanisms required to protect the respiratory tract from environmental injury. Macrophages (MΦ), which belong to a group of multipurpose defensive cells, provide the first line of defense against mucosal invasive pathogens. The present study was performed to investigate, whether the herbal medicinal product has anti-inflammatory or anti-oxidative effects on pro-inflammatorily activated MΦ or after oxidative stress induction. Special attention should be payed to elucidate the effects of *A. officinalis* on the mechanism of intracellular defense as well as on migratory capacity of the MΦ.

**Results:**

Treatment of PMA-differentiated human THP-1 MΦ with Phytohustil^®^ increased their viability without affecting the cell number. Phytohustil^®^ or root extracts of *A. officinalis* (REAo) – an active component of Phytohustil^®^ – were able to protect human MΦ against H_2_O_2_-induced cytotoxicity and H_2_O_2_-induced ROS production. Phytohustil^®^, REAo or diclofenac used as anti-inflammatory reference substance, inhibited the LPS-induced release of tumor necrosis factor-alpha (TNF-α) as well as of IL6 in MΦ. Treatment with Phytohustil^®^, its excipients or REAo did not impair the mitochondrial membrane potential (MMP). Finally, Phytohustil^®^ and REAo activated the migratory capacity of MΦ.

**Conclusion:**

The present *in vitro* investigations indicate protective, i.e., anti-oxidative and anti-inflammatory effects of REAo and Phytohustil^®^, additionally improving the migratory capacity of MΦ. These antiinflammatory effects were similar or even better than diclofenac. Thus, our data support and may explain the positive effect of Phytohustil^®^ observed in patients during the therapy of inflamed buccal mucosal membranes or treatment of cough.

## Introduction

*Althaea officinalis* L. (Malvaceae), also called marshmallow, is known as a medicinal plant from ancient time for the treatment of the irritation of laryngopharyngeal mucosa and hence associated dry cough. Many compounds have been extracted from *A. officinalis*, including starch (25–35%), pectins (11%), saccharose (10%), mucilage (5%), flavonoids, caffeic acid, p-coumaric acid, isoquercitrin, coumarins, phytosterols, tannins, etc., as well as many amino acids (Gudej, 1991; Bradley(ed.), 1992). Root extract of *Althaea officinalis* (REAo) contains water-miscible polysaccharides (acidic polysaccharides), mostly galacturorhamnans, arabinans, glucans, and arabinogalactans (Capek et al., 1987). The common oral use of REAo against dry cough caused by pharyngeal and mucosal irritation, is related to the bio-adhesive properties of the polysaccharides to the epithelial mucosa, which protects the cells from mechanical irritations and microbial invasion (Sendker et al., 2017). However, phytochemical investigations indicating the presence of bioactive low molecular weight compounds, as flavonoid-O-sulfoglycosides, and limiting the effects of REAo only to the mucilaginous effects of high molecular weight polysaccharides is not enough to explain the properties of *A. officinalis* (Sendker et al., 2017). O-sulfopolysaccharides are involved in the formation and regulation of the extracellular matrix (ECM) in the mucosal tissue (Sendker et al., 2017). This connection can trigger cell-matrix interactions and subsequent migration, cytokine signaling, as well as leukocyte activation in both, normal and pathological conditions (Korpos et al., 2010). The migration of MΦ, is greatly influenced by the composition of the local ECM, affecting both, the persistence and directionality of migration *in vivo* (Wang et al., 2006; Korpos et al., 2010). Transient recruitment and migration of polymorphonuclear leukocytes (PMNs), followed by MΦ accumulation is the host response to tissue injury or infection, characterized by the local production of inflammatory mediators, such as cytokines (Nathan, 2006). A special function in the regulation of ECM, especially in wound healing and inflammation, is attributed to hyaluronic acid (Sendker et al., 2017). It has recently been published that REAo inhibited the enzymatic activity of human hyaluronidase-1 expressed on the cell wall surface of *Escherichia coli* F470 bacteria and reduced its adhesive capacity on the ECM (Sendker et al., 2017). High molecular weight (>20 kDa) hydrophilic hyaluronic acid exerts anti-inflammatory effects by impairing the migration of leukocytes and MΦ, induction of cell proliferation and differentiation (Heldin, 2003). After migration, tissue-resident MΦ ingest bacteria, dead cells and recognize LPS, that stimulates the synthesis and secretion of pro-inflammatory cytokines, such as TNF-α, IL6, IL-1β etc. (Bochsler et al., 1993). Secretion of cytokines is an important component of host defense, allowing the immune system to detect and respond to small quantities of LPS in the early stages of bacterial infection, but anti-inflammatory agents are necessary to limit the cytokine hypersecretion during the resolution of the inflammation (Adams and Czuprynski, 1990). Many studies confirm that inflammation and oxidative stress are interdependent and interconnected processes. Inflammatory cells like MΦ release a number of ROS at the site of inflammation triggering oxidative damage and enhancing pro-inflammatory responses (McGarry et al., 2018). The balance of intracellular ROS is extremely important in maintaining normal physiology and cellular integrity. While the mitochondrial respiratory chain is the major component which cells use to produce intracellular ROS, cells reduce harmful excessive ROS via anti-oxidant enzymes such as catalase, superoxide dismutase, glutathione peroxidase, and glutathione reductase (Tan et al., 2016). Anti-oxidant properties of REAo have been described. Marshmallow exhibited strong total antioxidant activity, as well as effective reducing power, free radical/superoxide anion radical scavenging, and metal chelating activities (Elmastas et al., 2004), i.e., such extracts may be involved in the resolution of inflammation via anti-oxidative activity and phagocytosis regulation (Elmastas et al., 2004).

Phytohustil^®^ an herbal medicinal product containing REAo is commonly used for the treatment of mucous membrane irritations in the mouth and throat and the dry cough associated with this. The aim of this study was to investigate *in vitro* in human MΦ the beneficial anti-inflammatory and anti-oxidative properties of the well-known product Phytohustil^®^ compared to its major components, the REAo.

## Materials and Methods

### Cell Culture

The *in vitro* experiments were performed using the THP-1 (human acute monocytic leukemia) cell line (DSMZ GmbH, Braunschweig, Germany), cultured in RPMI complete medium [90% RPMI-1640 (PAA GmbH, Cölbe, Germany), 10% fetal bovine serum (FBS, PAA GmbH); 100 U/ml penicillin; 0.1 mg/ml streptomycin (PAA GmbH)].

### Tested Substances

Steigerwald Arzneimittelwerk GmbH (Steigerwald, Darmstadt, Germany) provided STW42-H marshmallow (REAo) as active constituent. REAo was prepared by maceration of the roots of *A. officinalis* in purified water (dry REAo batch-No. 14-0450; lot 00000816334) with a drug-extract-ratio (DER) of 3-9:1 after drying. 100 g Phytohustil^®^ cough syrup (Bayer AG, Leverkusen, Germany; batch-No. 730041; shelf life 2019/12) contains 35.6 g liquid REAo as active ingredient at a DER 19.5-23.5:1 according to DAC (German Drug Codex) 1999. Steigerwald Arzneimittelwerk GmbH also provided excipients, ethanol, propyl-4-hydroxybenzoate EP, methyl-4- hydroxybenzoate EP, sucrose. The concentration of Phytohustil^®^ was calculated in μg/ml dry extract.

The REAo was previously characterized and published (Sendker et al., 2015, 2017; Fink et al., 2018). A representative chromatogram of the REAo batch-No. 14-0450 is shown in the Supplementary Figure S1. Diclofenac sodium salt (Merck/Sigma-Aldrich Chemie GmbH, CAS, 15307-79-6) was used as an anti-inflammatory reference substance.

### Measurements of the Viability and Survival of Human MΦ

THP-1 cells (3 × 10^4^) seeded in 100 μl medium/well in 96-well plates (Falcon^TM^, BD Bioscience, Heidelberg, Germany) were incubated overnight in RPMI complete medium, and differentiated into MΦ by incubation with of 0.1 μg/ml phorbol-12-myristate-13-acetate (PMA, Merck/Sigma-Aldrich Chemie GmbH, Munich, Germany) for 3–5 days; afterward the medium was changed and different concentrations of the REAo, Phytohustil^®^ or its excipients were added. After 48 h treatment viability and cell number were measured as described below.

As a control (=100% viability), we used cells cultured with medium alone (∼ untreated control). Cell viability was assessed using PrestoBlue^®^ reagent (Fisher Scientific GmbH, Schwerte, Germany). PrestoBlue^®^ was directly added to the cells (into the culture medium) at a final concentration of 10% and measured according to the manufacturer’s specifications. Results were expressed in % of viability (OD_570__nm/__600__nm_ of samples × 100/OD_570__nm/__600__nm_ of untreated control). After the PrestoBlue^®^ reaction, the cells were fixed with 4% PFA/PBS and stained with crystal violet (Merck/Sigma-Aldrich Chemie GmbH) solution (0.04% crystal violet in 4% ethanol [v/v]) and washed; afterward the cells were lysed in a 1% sodium dodecyl sulfate (SDS, Merck/Sigma-Aldrich Chemie GmbH) solution. The crystal violet absorbance was measured at 595 nm (reference 655 nm) to spectrophotometrically determine the total cell number.

### Determination of the Protective Effects Against H_2_O_2_-Induced Cytotoxicity

The protective effect of the REAo, Phytohustil^®^ or its excipients against cytotoxicity induced by H_2_O_2_ treatment was determined using the PrestoBlue^®^ viability assay (Fisher Scientific GmbH) and crystal violet cell quantification assay as described above. In detail, 3 × 10^4^ THP-1 cells were seeded in 100 μl medium/well using 96-well plates (Falcon^TM^, BD Bioscience) and differentiated with PMA in MΦ, the medium was changed and the MΦ were pre-treated for 48 h with non-cytotoxic concentrations of the REAo, Phytohustil^®^ or its excipients. Afterward, the MΦ were treated with or without 5 mM H_2_O_2_ (3 h) and quantification of viability and cell number was performed.

### Determination of the Mitochondrial Membrane Potential (MMP, ΔΨm)

Mitochondrial membrane potential was measured in 3 × 10^4^ PMA-differentiated MΦ as described above by using 10 μM of the fluorescent mitochondrial dye JC-10 (Biomol GmbH, Hamburg, Germany) in black Lumox multi well plates (Sarstedt AG & Co., Nümbrecht, Germany). After treatment with the REAo, Phytohustil^®^ or its excipients, the cells were incubated (5–15 min) in serum-free medium containing JC-10 dye loading solution (according to the manufacturer’s protocol). JC-10 accumulates in mitochondria, selectively generating an orange J-aggregate emission profile (590 nm) in healthy cells. However, upon cell injury, as membrane potential decreases, JC-10 monomers are generated, resulting in a shift to green emission (525 nm) to detect subtle changes in MMP. The MMP was measured, considering the fluorescence intensity ratio or relative fluorescence units (RFU), of changes measured at 490 nm excitation/525 nm emission (FITC, green) divided by 540 nm excitation/590 nm emission (TRITC, red/orange), using the Cytation^TM^ 3 Cell Imaging Multi-Mode Reader (BioTek Instruments). The MMP RFU_490__/__525_/RFU_540__/__590_ was normalized against the Hoechst 33342 RFU (RFU of cell nuclei) quantified at 350 nm excitation/461 nm emission. Treatment with 12.5 mM H_2_O_2_ was used as control of mitochondrial membrane depolarization.

### Determination of Anti-inflammatory Effects

The release of TNF-α or IL6 was determined using enzyme-linked immunosorbent assay (ELISA). In detail, 3 × 10^4^ PMA-differentiated MΦ were seeded in 100 μl medium/well using 96-well plates (Falcon^TM^, BD Bioscience); thereafter, the medium was changed and the MΦ were pre-treated for 48 h with different concentrations of REAo, Phytohustil^®^ or its excipients contained in the corresponding concentrations of Phytohustil^®^. Afterward, the cells were activated for 3 h with 0.01 μg/ml (TNF-α experiments) or 1.0 μg/ml (IL6 experiments) ultrapure lipopolysaccharide (LPS-EB) from *E. coli* O111:B4 (Cayla – InvivoGen Europe, Toulouse France) – that is only recognized by toll-like receptor 4 (TLR4). Human TNF-α or IL6 were determined in the culture medium using the assay Duo Set ELISA Development kit (R&D Systems Europe, Ltd., Abingdon, United Kingdom) following the manufacturer’s instructions. Afterward the cells were fixed with 4% PFA/PBS and stained with crystal violet and the absorbance was measured as described above. The TNF-α or IL6 OPD absorbance (490 nm and 655 nm reference) was normalized to the crystal violet absorbance. The results were expressed as % of the released TNF-α or IL6 after stimulation with LPS which was considered as 100% release. Additionally, diclofenac sodium salt was used as anti-inflammatory reference substance in our experimental setting (Rupasinghe et al., 2015; Bonaterra et al., 2019).

### Determination of Cellular Reactive Oxygen Species (ROS)

The production of ROS was measured by detecting the fluorescent intensity of the oxidant-sensitive probe 2′-7′-dichlorofluorescin diacetate (DCFDA, Merck/Sigma-Aldrich Chemie GmbH). ROS were measured in 3 × 10^4^ PMA-differentiated MΦ after pre-treatment with REAo, Phytohustil^®^ or its excipients, and afterward with 12.5 mM H_2_O_2_ during 3 h. The production of ROS was detected by using 10 μM of the fluorescent DCFDA incubated during 30 min. Total ROS were quantified, considering the fluorescence intensity RFU, measured at 495 nm excitation/529 nm emission and normalized against the Hoechst 33342 RFU (RFU of cell nuclei) measured at 350 nm excitation/461 nm emission using the Cytation^TM^ 3 Cell Imaging Multi-Mode Reader (BioTek Instruments).

### Determination of the Migratory Capacity of MΦ

The migratory capacity of MΦ was determined using a scratch assay in 24-well plates; scratches in the cell monolayer at the bottom of the well were made with pipette tips. Then the medium was changed and the cells were exposed to different concentrations (300–500 μg/ml) of REAo, Phytohustil^®^ or its excipients (24 h). The scratch was photographed at time 0 and after 24 h, using an inverted microscope Axiovert 135, equipped with motorized stage and a digital AxioCam MRc camera (Carl Zeiss AG, Oberkochen, Germany). Effects on MΦ migration were plotted as a percentage of closure of the scratch (% of the scratch closure = [(Δt) × 100%]/At 0 h; where “At 0 h” is the area of the scratch measured immediately after scratching, “At 24 h” indicates the area of the scratch measured 24 h after scratching and Δt = At 0 h–At 24 h (Yue et al., 2010).

### Statistical Analyses

The SigmaPlot 12 software (Systat Software GmbH, Erkrath, Germany) was used to carry out statistical analyses by the unpaired Student’s *t* test or Mann–Whitney *U*-test. Data was shown as mean + SEM. *p* < 0.05 was considered as statistically significant.

## Results

### Effect of Phytohustil^®^ and Root Extract of *A. officinalis* on the Viability of MΦ

**FIGURE 1:**
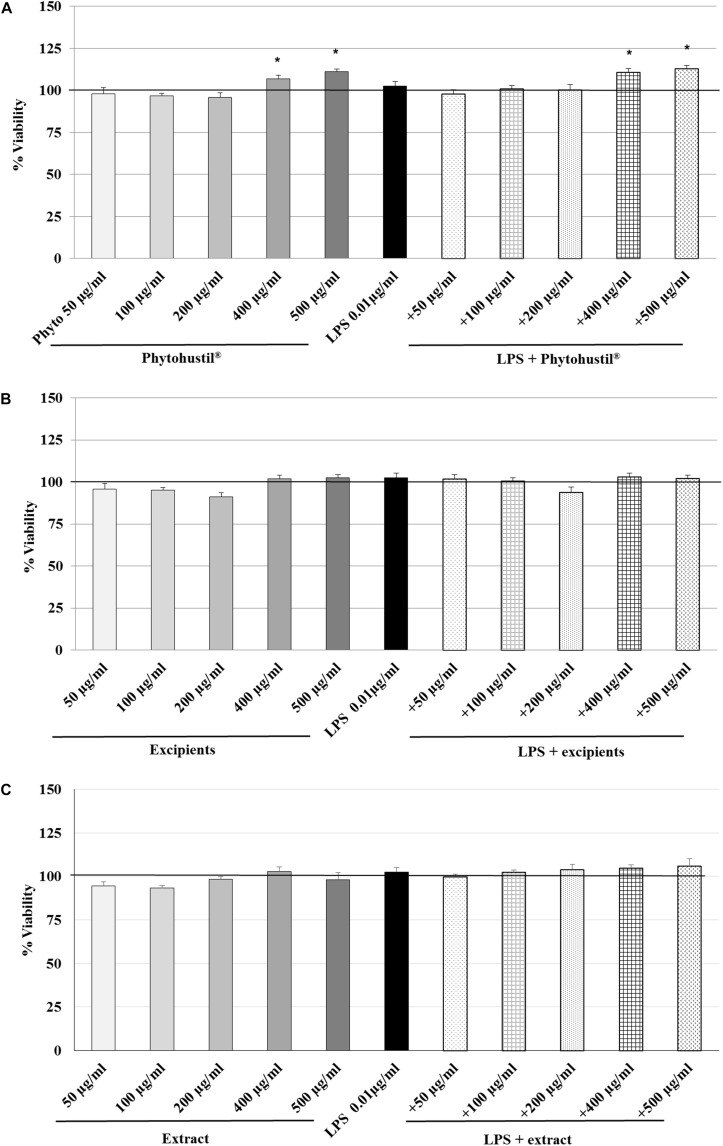
Effects of treatment (48 h) of human MΦ with Phytohustil^®^ **(A)**, its excipients **(B)** or **(C)** REAo (extract) – with or without LPS – on viability are shown. Values [in % viability of untreated control (∼100% viability)] are given as mean + SEM; *p < 0.05 (by *T*-TEST) significance vs. untreated control. *n* = 4–5 independent experiments.

First, we investigated the effect of 48 h treatment on the viability on MΦ using PrestoBlue^®^ cell viability reagent. PrestoBlue^®^ reactivity is based on resazurin, which functions as a cell viability indicator based on mitochondrial enzyme activity (Xu et al., 2015). In viable cells, resazurin is reduced to resorufin in cellular respiration by accepting electrons from NADPH, FADH, FMNH, NADH and cytochromes (Al-Nasiry et al., 2007) and may be used to measure mitochondrial activity. The treatment of MΦ with 0.01 μg/ml LPS did not affect their viability (Figures 1A–C). 400 and 500 μg/ml Phytohustil^®^ alone, significantly (*p* < 0.05) increased the viability by +7.0 to +11.0% in comparison with the negative untreated control (MΦ incubated with medium alone; = 100% viability) (Figure 1A). Pre-treatment of MΦ with 400 or 500 μg/ml Phytohustil^®^ and afterward treatment with 0.01 μg/ml LPS significantly (*p* < 0.05) increased the viability by 10.7% or 13.0% (Figure 1A). The pre-treatment of MΦ with Phytohustil^®^’s excipients or REAo with or without LPS did not affect the cell viability (Figures 1B,C) and the cell quantity determined by CV (not shown).

### Protective Effects of Phytohustil^®^ or REAo Against H_2_O_2_-Induced Cytotoxicity in MΦ

**FIGURE 2:**
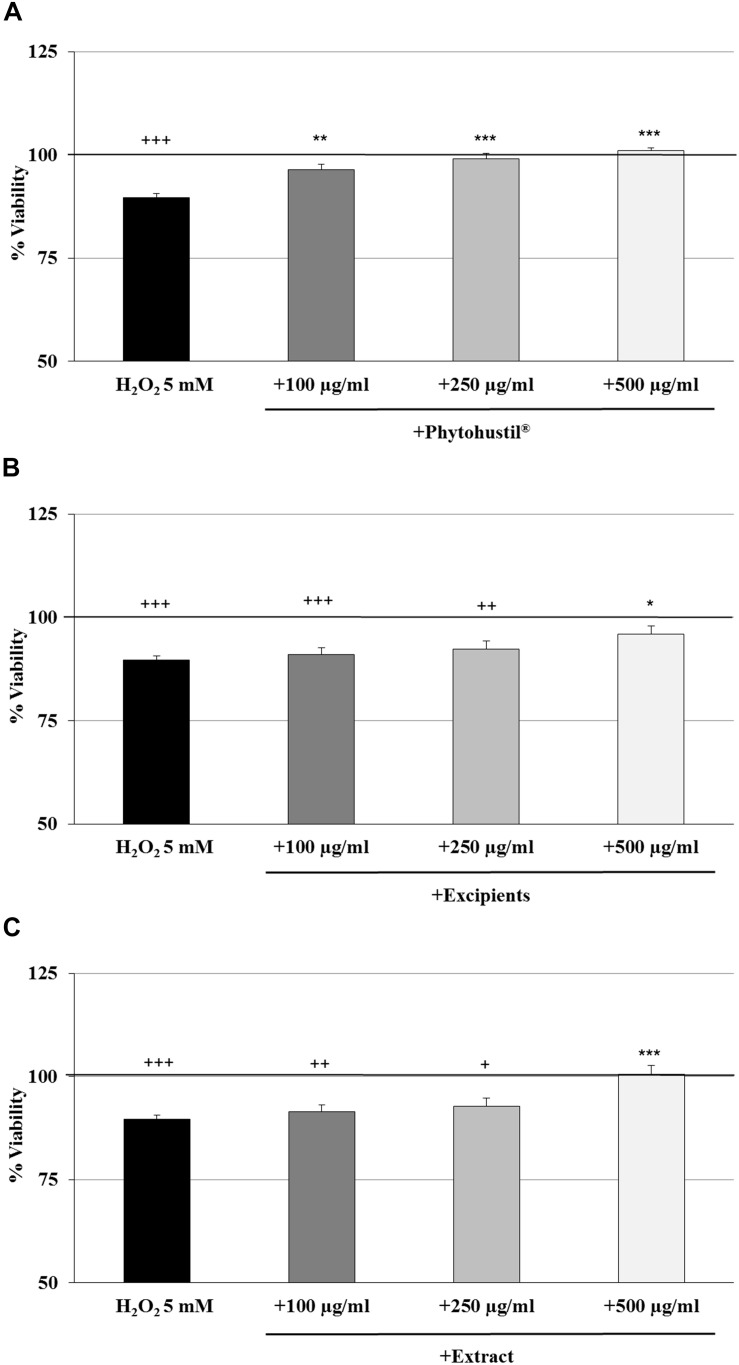
Protective effects of Phytohustil^®^, its excipients or REAo against H_2_O_2_–induced cytotoxicity by pre-treatment (48 h) of human MΦ with Phytohustil^®^ **(A)**, its excipients **(B)** or **(C)** REAo (extract) – with or without H_2_O_2_ – on the viability are shown. Values [in % viability of untreated control (∼100% viability)] are given as mean + SEM; *p < 0.05, **p < 0.01, ***p < 0.01 significance vs. H_2_O_2_ treatment; ^+^*p* < 0.05, ^++^*p* < 0.01, ^+++^*p* < 0.001 significance vs. untreated control as 100% viability (by *T*-TEST); *n* = 4–5 independent experiments.

**FIGURE 3:**
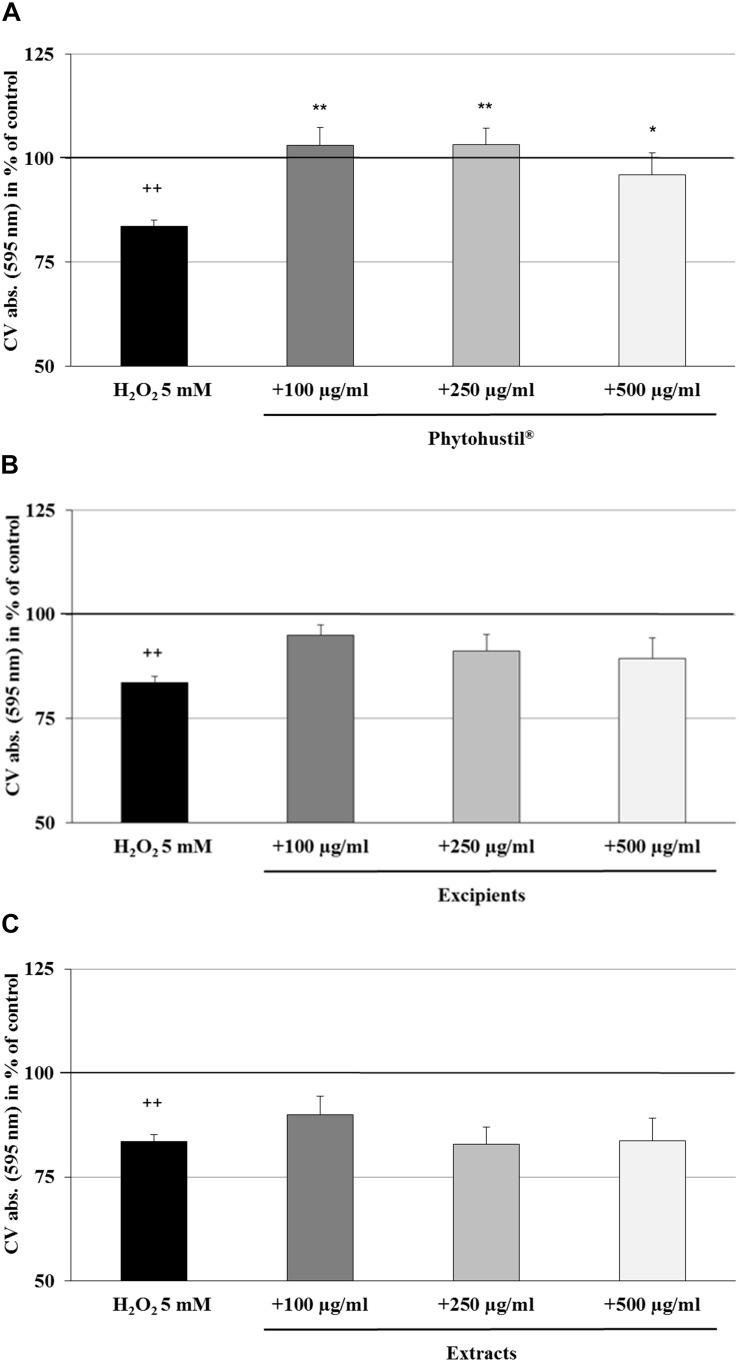
Protective effects of Phytohustil^®^, its excipients or REAo against H_2_O_2_–induced cytotoxicity by pre-treatment (48 h) of human MΦ with Phytohustil^®^ **(A)**, its excipients **(B)** or **(C)** REAo (extract) with or without H_2_O_2_- on cell quantity are shown. Values [in % CV absorbance of untreated control (∼100%)] are given as mean + SEM; *p < 0.05, **p < 0.01 significance vs. H_2_O_2_ treatment; ^++^*p* < 0.01 significance vs. untreated e control (∼ 100%) by *T*-TEST; *n* = 4–5 independent experiments.

We investigated the cytotoxic effects of H_2_O_2_ and found that 5 mM significantly (*p* < 0.001) inhibited the viability by 10.4% in comparison with the untreated control (Figures 2A–C). Pre-treatment (48 h) of MΦ with 100 μg/ml, 250 μg/ml, or 500 μg/ml Phytohustil^®^ led to a significant concentration-dependent inhibition of H_2_O_2_-induced cytotoxicity by 6.7% (*p* < 0.01), 9.5% (*p* < 0.001), and 11.4% (*p* < 0.001) in comparison to 3 h treatment with 5 mM H_2_O_2_ (Figure 2A). Pre-treatment of MΦ with excipients only at the concentration of 500 μg/ml, showed a significant (*p* < 0.05) 6.3% inhibition of H_2_O_2_-induced cytotoxicity (Figure 2B). Pre-incubation of MΦ with 500 μg/ml REAo significantly (*p* < 0.001) attenuated the 5 mM H_2_O_2_-mediated cytotoxicity by 10.9% (Figure 2C). After treatment of MΦ with 5 mM H_2_O_2_ we found a significantly (*p* < 0.01) decreased cell number by 16.4% when compared to the untreated control, considered as 100% of CV absorbance (as 100% cell quantity) (Figures 3A–C). Pre-treatment with 100 μg/ml, 250 μg/ml or 500 μg/ml Phytohustil^®^ significantly inhibited the H_2_O_2_-mediated decreasing cell number by 19.5% (*p* < 0.01), 19.7% (*p* < 0.01), and 12.4% (*p* < 0.05) (Figure 3A). Incubation of MΦ with excipients exhibited no cell number protecting effects against H_2_O_2_ induced-cytotoxicity (Figure 3B). Pre-treatment with REAo also showed no cell number protecting effects compared to H_2_O_2_-treated cells (Figure 3C).

### Effects of Phytohustil^®^ or REAo on the Mitochondrial Membrane Potential (MMP) in MΦ

**FIGURE 4:**
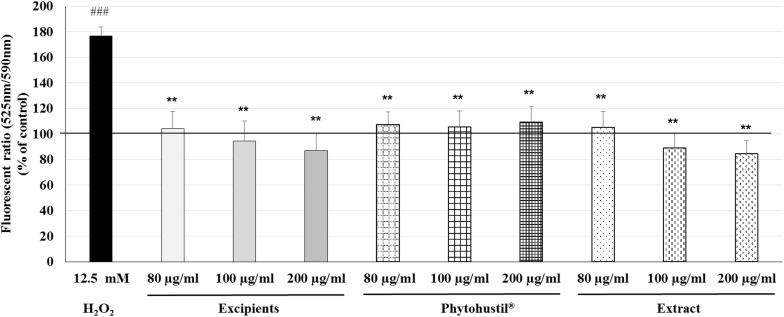
Effects of Phytohustil^®^, its excipients or REAo on mitochondrial membrane potential (MMP, ΔΨm) state in human MΦ. Values in % Fluorescence = {[Sample (FITC _525__nm_/TRITC _590__nm_)/Hoechst _461__nm_]* 100}/untreated control (FITC _525__nm_/TRITC _590__nm_)/Hoechst _461__nm_] are given as mean + SEM; **p < 0.01 (by *T*-TEST) significance vs. H_2_O_2_-treated cells; ^###^*p* < 0.001 vs. untreated control (100%); *n* = 3–4 independent experiments.

According to the results observed after quantification of cell viability, we investigated the effects of Phytohustil^®^, its excipients or REAo on the MMP. We utilized the JC-10 dye, which is useful for determining the MMP by fluorescence microscopy. H_2_O_2_ (12.5 mM) is used as a control, which significantly (*p* < 0.001) increased the MMP depolarization by 76.7% compared to untreated control (∼ 100% MMP integrity) (Figure 4). Treatment (80, 100, or 200 μg/ml; 48 h) of MΦ with Phytohustil^®^, its excipients or REAo did not affect the MMP compared to the untreated control (Figure 4).

### Protective Effects of Phytohustil^®^ or REAo Extract Against H_2_O_2_-Induced on ROS Production in MΦ

**FIGURE 5:**
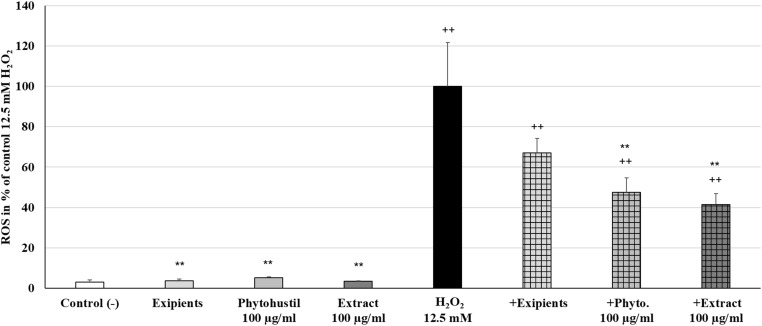
Protective effects of Phytohustil^®^, its excipients or REAo against H_2_O_2_–induced reactive oxygen species (ROS) production in human MΦ, using the cell permeant reagent 2′,7′ –dichlorofluorescin diacetate. Values in% Fluorescece = [Sample (FITC _529__nm__/_Hoescht _461__nm_)* 100]/(untreated control (FITC _529__nm__/_Hoescht _461__nm_) are given as mean + SEM; significance (by *T*-TEST) ^++^*p* < 0.01 vs. untreated control; **p < 0.01 vs. H_2_O_2_ 12.5 mM; *n* = 6 independent experiments.

Reactive oxygen species are generated during mitochondrial oxidative metabolism. When ROS rise above the antioxidant defenses, because of a decrease in the cellular antioxidant capacity or an increase in ROS levels, oxidative stress occurs. Incubation of MΦ with Phytohustil^®^ (100 μg/ml), its excipients or REAo (100 μg/ml) did not affect the ROS level compared to untreated control (Figure 5). Treatment with H_2_O_2_ (12.5 mM) significantly (*p* < 0.01) increased the ROS production by 96.9% compared to untreated control (Figure 5). Pre-treatment of MΦ with 100 μg/ml Phytohustil^®^ or 100 μg/ml REAo and additional incubation with 12.5 mM H_2_O_2_ significantly (*p* < 0.01) inhibited the ROS production by 52.4% or by 58.7%. compared to MΦ stimulated with 12.5 mM H_2_O_2_ alone (Figure 5).

### Protective Effects of Phytohustil^®^ or REAo on LPS-Induced TNF-α Release by MΦ

**FIGURE 6:**
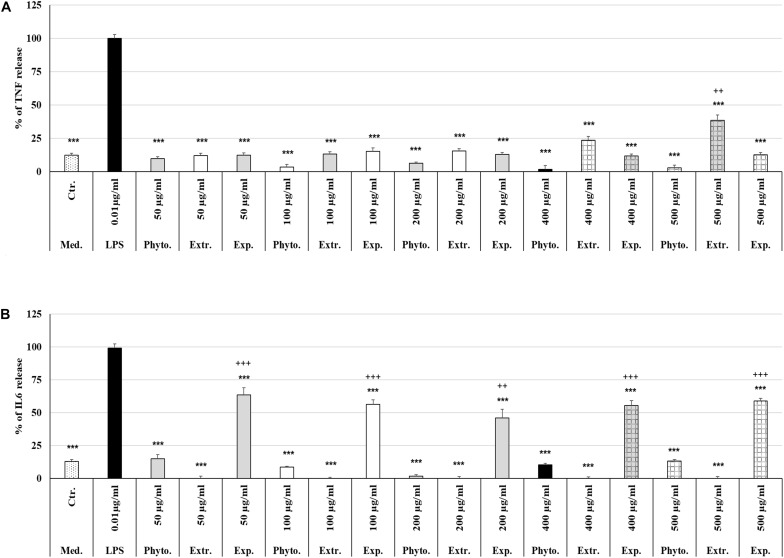
Effects of Phytohustil^®^, its excipients or REAo on TNF-α or IL6 release. **(A)** Effects of treatment (48 h) of human MΦ with Phytohustil^®^, REAo (Extr.) or excipients (Exp) on TNF-α release. **(B)** On IL6 release. Untreated control (Ctr.). Data are given as mean + SEM; ***p < 0.001 (by *T*-TEST) significance vs. LPS treated human MΦ (∼100%); ^++^*p* < 0.01, ^+++^*p* < 0.01, Phytohustil^®^ vs. untreated control (ctr.) *N* = 4–5 independent experiments.

**FIGURE 7:**
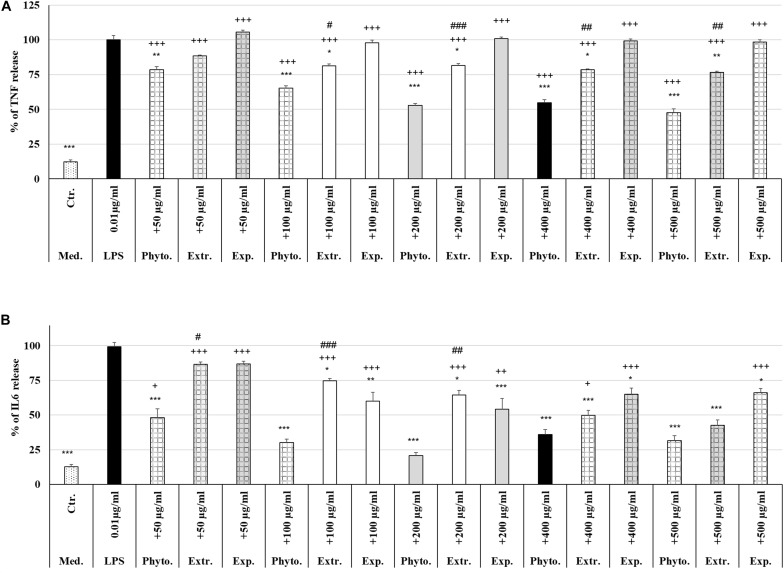
Protective effects of Phytohustil^®^, its excipients or REAo against LPS–induced TNF-α or IL6 release. **(A)** Effects of pre-treatment (48 h) of human MΦ with Phytohustil^®^ (Phyto.), REAo (Extr.) or Phytohustil^®^’s excipients – with LPS (1 μg/ml; 3 h) on TNF-α release. **(B)** Effects on IL6 release Data are given as mean + SEM; *p < 0.05, **p < 0.01, ***p < 0.001 (by *T*-TEST) significance vs. 3 h LPS treatment taken as 100%; ^+^*p* < 0.05, ^+ +^ *p* < 0.01, ^+++^*p* < 0.001, Phytohustil^®^ vs. untreated control (ctr.) and ^#^*p* < 0.05, ^##^*p* < 0.01, ^###^*p* < 0.001 Phytohustil^®^ vs. REAo (B). *N* = 3–4 independent experiments.

Treatment (48 h) of MΦ with different concentrations (50 μg/ml, 100 μg/ml, 200 μg/ml, 400 μg/ml, 500 μg/ml) of Phytohustil^®^, its excipients or REAo alone did not affect the TNF-α release compared to untreated control, except 500 μg/ml REAo increased significantly (*p* < 0.01) the TNF-α release compared to untreated control (Figure 6A). Incubation of MΦ with LPS (0.01 μg/ml) induced a significantly (*p* < 0.001) increased TNF-α release of 87.8% compared to untreated control (Figures 6A, 7A). Furthermore, MΦ were pre-treated (48 h) with Phytohustil^®^ (50–500 μg/ml), its excipients or REAo and additionally activated with LPS (0.01 μg/ml; 3 h) to measure TNF-α release. Pre-treatment with Phytohustil^®^ significantly inhibited LPS-induced TNF-α release by 21.6% (50 μg/ml, *p* < 0.01), 34.8% (100 μg/ml, 0.001), 47.3% (200 μg/ml, *p* < 0.001), 45.2% (400 μg/ml, *p* < 0.001), and 52.4% (500 μg/ml, *p* < 0.001) in comparison with LPS-treated MΦ (Figure 7A). Pre-treatment with REAo significantly inhibited the TNF-α release by 18.8% (100 μg/ml, 0.05), 18.3% (200 μg/ml, *p* < 0.05), 21.5% (400 μg/ml, *p* < 0.01), and 23.3% (500 μg/ml, *p* < 0.01) in comparison with LPS-treated MΦ (Figure 7A). In general, the comparison of the anti-inflammatory effect of different concentrations of Phytohustil^®^ and corresponding concentrations of REAo revealed significantly higher inhibitory effects on the LPS-induced TNF-α release by Phytohustil^®^ by 16.1% (*p* < 0.05; 100 μg/ml), 28.9% (*p* < 0.001; 200 μg/ml), 23.7% (*p* < 0.01; 400 μg/ml), and 29.1% (*p* < 0.01; 500 μg/ml) (Figure 7A). Incubation of MΦ with different concentrations of excipients did not inhibit the LPS-induced TNF-α release and was significantly (*p* < 0.001) higher than the untreated control (Figure 7A).

### Protective Effects of Phytohustil^®^ or REAo on LPS-Induced IL6 Release by MΦ

In order to confirm the anti-inflammatory effects observed by Phytohustil^®^ and REAo we determined the inhibition of the IL6 release of LPS-activated MΦ. Treatment of MΦ with REAo or Phytohustil^®^ (50, 100, 200, 400, or 500 μg/ml) alone did not affect the IL6 release compared to untreated control (Figure 6B). In contrast, treatment with Phytohustil^®^’s excipients significantly (*p* < 0.001) induced the release of IL6 compared to untreated control (Figure 6B). Incubation of MΦ with LPS (1 μg/ml, 3 h) induced a significantly (*p* < 0.001) increased IL6 release of 86.3% compared to untreated control (Figures 6B, 7B). Pre-treatment with Phytohustil^®^ significantly inhibited LPS-induced IL6 release by 51.2% (50 μg/ml, *p* < 0.001), 69.0% (100 μg/ml, *p* < 0.001), 78.5% (200 μg/ml, *p* < 0.001), 63.3% (400 μg/ml, *p* < 0.001), 67.7% (500 μg/ml, *p* < 0.001) in comparison to LPS-treated MΦ (Figure 7B). Pre-treatment with REAo significantly inhibited the IL6 release by 24.6% (100 μg/ml, *p* < 0.05), 34.7% (200 μg/ml, *p* < 0.05), 49.3% (400 μg/ml, *p* < 0.001), and 56.46% (500 μg/ml, *p* < 0.001) in comparison with LPS treated MΦ (Figure 7B). In general, the comparison of the anti-inflammatory effect of different concentrations of Phytohustil^®^ and corresponding concentrations of REAo revealed significantly higher inhibitory effects on the LPS-induced IL6 release by Phytohustil^®^ 38.5% (*p* < 0.05, 50 μg/ml) and 44.5% (*p* < 0.001; 100 μg/ml) and 43.7% (*p* < 0.01; 200 μg/ml) but not by 400 μg/ml or 500 μg/ml (Figure 7B).

Incubation of MΦ with excipients corresponding to 100 μg/ml, 200 μg/ml, 400 μg/ml, or 500 μg/ml of Phytohustil^®^ significantly inhibited the IL6 release by 39.9% (*p* < 0.01), 45.0% (*p* < 0.001), 34.2% (*p* < 0.05), or 33.1% (*p* < 0.05) in comparison with LPS treated MΦ (Figure 7B).

### Comparison of Anti-inflammatory Properties of Phytohustil^®^ or REAo With Diclofenac

**FIGURE 8:**
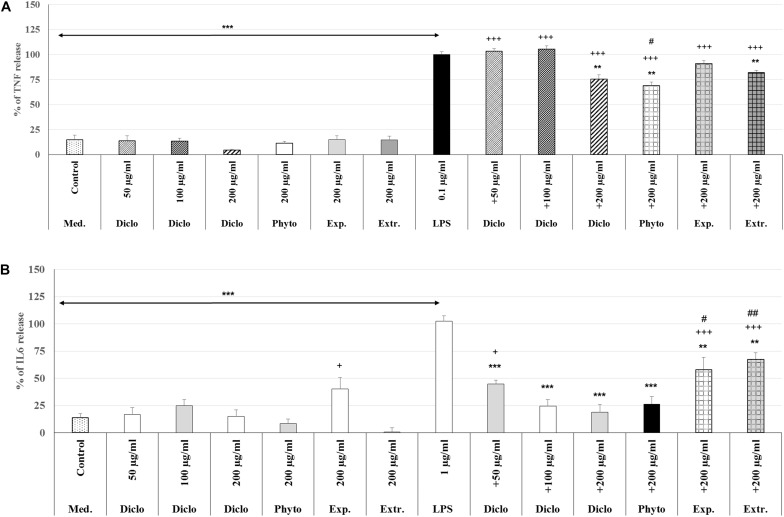
Anti-inflammatory effects of Phytohustil^®^ (Phyto.), its excipients (Exp), or REAo (Extr.) against **(A)** LPS–induced Tumor Necrosis Factor (TNF-α) and **(B)** Interleukin-6 (IL6) release (measured by ELISA) in human MΦ compared to diclofenac sodium salt (Diclo) treatment. Medium control (med. control). Data are given as mean + SEM; **p < 0.01, ***p < 0.001 (by *T*-TEST) significance vs. 3 h LPS treatment taken as 100%; ^+^*p* < 0.05, ^+++^*p* < 0.001 vs. control and ^#^*p* < 0.05, ^##^*p* < 0.01 vs. 200 μg/ml diclofenac. *N* = 5–7 independent experiments.

The treatment with 0.1 μg/ml LPS (3 h) induced a significant 85.0% (*p* < 0.001) increase of the TNF-α release compared to the untreated control (Figure 8A). Pre-treatment with 200 μg/ml diclofenac significantly inhibited LPS-induced TNF-α release by 24.6% (*p* < 0.01) compared to LPS. Pre-treatment with 200 μg/ml Phytohustil^®^ as well as REAo showed an inhibition of 31.1% (*p* < 0.01) and 17.8% (*p* < 0.01) compared to LPS. Whereas Phytohustil^®^ excipients did not inhibit the LPS-induced TNF-α release when compared to LPS (Figure 8A). Our results also indicate that treatment with 200 μg/ml Phytohustil^®^ was significantly (*p* < 0.05) 6.7% more effective than 200 μg/ml diclofenac, whereas TNF-α release of REAo was similar to diclofenac (Figure 8A). Neither diclofenac nor Phytohustil^®^, its excipients or REAo alone did induce TNF-α release (Figure 8A). Treatment (3 h) with 1.0 μg/ml LPS resulted in a significant 89.0% (*p* < 0.001) increase of the IL6 release compared to the untreated control (Figure 8B). Pre-treatment with 200 μg/ml diclofenac significantly (*p* < 0.001) inhibited the LPS-induced IL6 release by 83.6%. Pre-treatment with 200 μg/ml Phytohustil^®^ revealed similar anti-inflammatory effects like 200 μg/ml diclofenac (Figure 8B). Pre-treatment with 200 μg/ml REAo or excipients significantly inhibited LPS-induced IL-6 release. However, this inhibition was significantly lower than 200 μg/ml Phytohustil^®^ or diclofenac (Figure 8B). At 200 μg/ml neither diclofenac nor Phytohustil^®^ or REAo alone induced an IL6 release. However, incubation with 200 μg/ml excipients alone significantly 26.6% (*p* < 0.05) induced an IL6 release (Figure 8B).

### Stimulatory Effects of Phytohustil^®^ or REAo on the Migratory Capacity of MΦ

**FIGURE 9:**
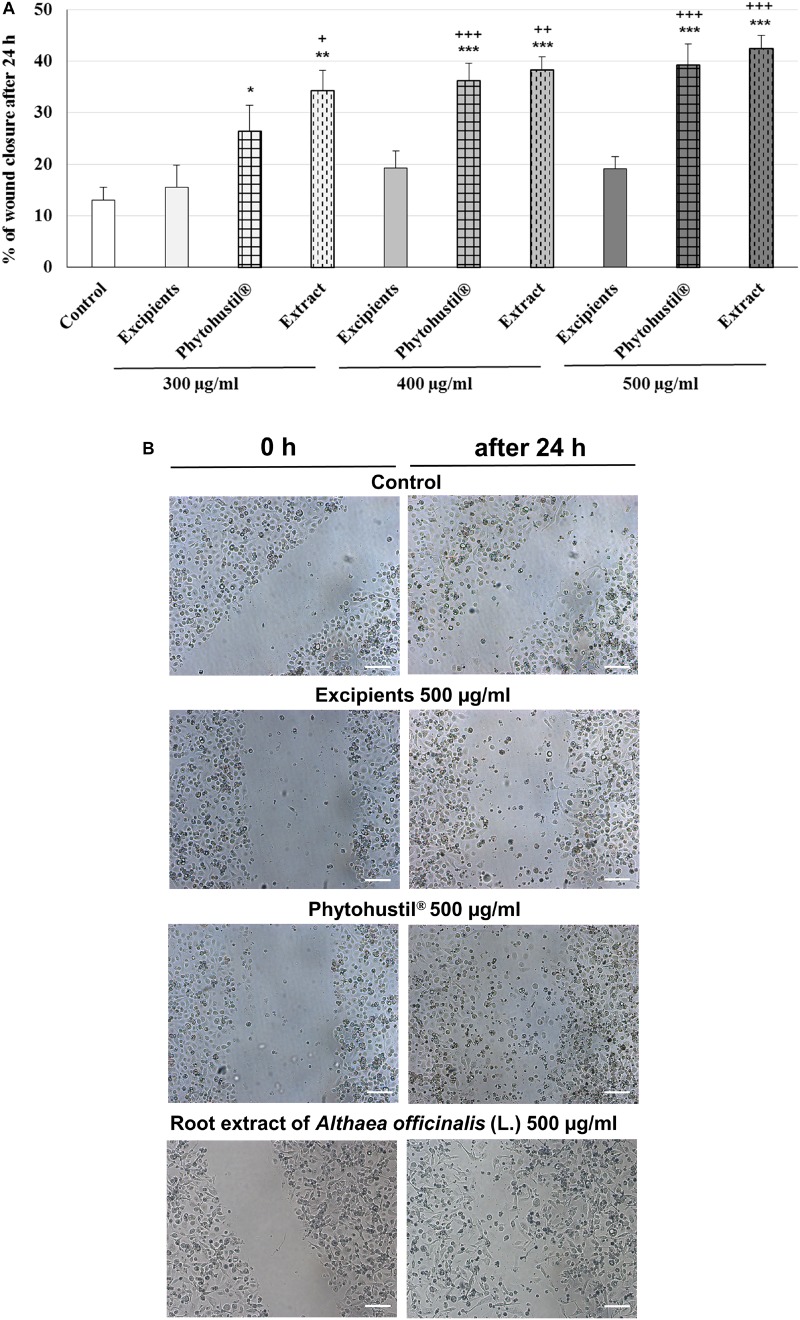
Stimulatory effects of Phytohustil^®^ or REAo on the migratory capacity of human MΦ. **(A)** Quantification of cell migration of human MΦ after scratching. MΦ were treated with Phytohustil^®^ its excipients or REAo for 24 h or medium alone (untreated control). The results are expressed in% of scratch closure as mean + SEM. *T*-TEST vs. untreated control, *p < 0.05, **p < 0.01, ***p < 0.001 and vs. excipients ^+^*p* < 0.05, ^++^*p* < 0.01, ^+++^ *p* < 0.001. **(B)** Representative images of the wound effect after 24 h. *N* = 4 independent experiments. Scale bar: 100 μm.

We investigated the migratory capacity of MΦ after treatment with REAo, Phytohustil^®^ or its excipients (300, 400, or 500 μg/ml) using the scratch assay. The results indicate that the treatment with Phytohustil^®^ significantly stimulated the migratory capacity and scratch closure after 24 h treatment in a concentration-dependent manner by 2.0-fold (300 μg/ml, *p* < 0.05), 2.8-fold (400 μg/ml, *p* < 0.001), 3.0-fold (500 μg/ml, *p* < 0.001) compared to untreated control (Figures 9A,B). Treatment with 300 μg/ml, 400 μg/ml or 500 μg/ml REAo significantly stimulated the migratory capacity by 2.6-fold (*p* < 0.01), 2.9-fold (*p* < 0.001) or 3.2-fold compared to untreated control (Figures 9A,B). In contrast, the treatment with excipients did not stimulate the migratory capacity (Figures 9A,B).

## Discussion

The discovery of active natural products is of great interest for treatment of disease as dry cough due to irritation of the oral and pharyngeal mucosa. Marshmallow is well-known for its healing properties since ancient time. It has been reported that a water extract from roots of *A. officinalis* had stimulating effects on cell viability and proliferation of epithelial cells, but not on primary fibroblasts (Deters et al., 2010; Benbassat et al., 2014). We now found that Phytohustil^®^ increased the viability of MΦ, key components of the innate immune defense system, without revealing cytotoxic effects. In line with these findings, the REAo had positive effects on the viability of epithelial cells involved in the mucosal barrier (Deters et al., 2010). The regulation of the bioenergetic metabolism plays a central role in the physiology of MΦ, including mitochondria, which play an essential role in regulating the responses of MΦ to injury, pathogens, and inflammation in the tissues (Ravi et al., 2014). The mitochondrial pathway of apoptosis is mediated by disruption of the outer membrane and consequently the depolarization of the MMP (Gupta et al., 2009). Furthermore, MMP impacts directly the control of redox status, proliferation and cell death (Zorov et al., 2014). Normally, cells maintain stable levels of intracellular ATP and the MMP; this stability is a requisite for normal cell functioning (Zorov et al., 2014), a disruption of this homeostasis causes cell damage. We found, that treatment of MΦ with different concentrations of Phytohustil^®^, its excipients or REAo protect the MMP at levels of the untreated control. MMP is implicated in the role of mitochondria in cellular homeostasis, together with ROS generation (Zorov et al., 2014). ROS include a number of reactive molecules and free radicals derived from molecular oxidation, produced as byproducts during the mitochondrial aerobic respiration and has the potential to cause intracellular damage. Phagocytic cells like MΦ are also responsible for ROS production and play a major role in the activation of cell signaling cascades, including apoptosis (Gupta et al., 2009; Zorov et al., 2014). We performed experiments to investigate the possible protective properties of Phytohustil^®^ or REAo, against H_2_O_2_ -induced cytotoxicity and intracellular ROS production. We found an inhibition of H_2_O_2_–mediated decrease of the viability, when MΦ were pre-treated with Phytohustil^®^. Our findings are congruent with publications of others (Sadighara et al., 2012; Benbassat et al., 2014) showing that Phytohustil^®^ and REAo have antioxidant properties, stimulate anti-oxidative defense mechanisms and, thus, may protect against intracellular ROS increase, i.e., oxidative stress in MΦ. In contrast to non-mucosal tissues, the mucosal tissues have close contact with numerous and diverse commensal microorganisms, as well as pathogens, which can trigger pro-inflammatory responses. Therefore, inhibition of cytokine production is the common mechanism of action of anti-inflammatory compounds. Topical application of the REAo was shown to act anti-inflammatorily to UV-irradiated skin of rabbits (Beaune and Balea, 1966). Other *in vivo* experiments indicated a marginal immune-activating effect of extracts or purified marshmallow polysaccharides (Wagner, 1990). However, an ethanolic extract from marshmallow root, administrated orally, did not inhibit carrageenan-induced rat paw oedema (Mascolo et al., 1987). In differentiated MΦ, LPS induced a strong dose-dependent up-regulation of inflammatory cytokines, such as TNF-α and IL6 (Kato et al., 2004; Chen and Cheng, 2009), which can be taken as an *in vitro* test - similar to our MΦ - to investigate anti-inflammatory properties of drugs. We found that Phytohustil^®^ and the REAo, but not its excipients, inhibited the LPS-stimulated release of TNF-α and IL6 by MΦ, corroborating the anti-inflammatory and possible immunomodulatory properties of the REAo as described by others in neutrophils and monocytes (Scheffer and König, 1991). In general, cytokines produced by monocytes and MΦ (e.g., TNF-α, IL6) promote monocyte survival and differentiation, and thus, may explain the presence of large number of MΦ in a lesion (Mangan et al., 1993). We have performed our *in vitro* experiments using differentiated MΦ instead immature monocytes, because it is well-known that LPS-activated MΦ exhibit another response after pre-treatment with anti-inflammatory substances than monocytes (Mangan et al., 1993; Bonaterra et al., 2010). The results of our experiments showed that pre-treatment with Phytohustil^®^ or REAo inhibit the LPS-induced TNF-α- and IL6 release and corroborate the anti-inflammatory properties of REAo. These anti-inflammatory effects of the REAo and the commercially available product Phytohustil^®^ on differentiated MΦ are shown here for the first time.

The effect of incubation of MΦ with 200 μg/ml Phytohustil^®^ or REAo on LPS-induced TNF-α release was similar to the effects of diclofenac, which served as reference substance. The inhibitory effects of 200 μg/ml Phytohustil^®^ on the IL6 release were comparable to the effect of 100 or 200 μg/ml diclofenac, but not to those of Phytohustil^®^’exipients or REAo. Consequently, properties of Phytohustil^®^ and the REAo may have special impact on the resolution of the mucosal inflammation and alleviate the irritated oral and pharyngeal mucosa, which can explain its known pharmacological effects and clinical efficacy.

Monocyte migration plays an important role in physiological and pathological processes that include wound healing, repairing mucosal damage and resolution of inflammation (Leoni et al., 2015). During the infection process, e.g., after mucosal injury, monocytes are initially recruited from the blood stream into the mucosal injury/wound site (Chanput et al., 2010; Wynn et al., 2013; Crane et al., 2014) and rapidly differentiate into so-called “wound-associated MΦ” (Chanput et al., 2010; Wynn et al., 2013; Crane et al., 2014). Human THP-1 cells, have widely been used as a model to study the immune response capacity of monocytes and MΦ, because of similarities in their responses, when compared to monocytes isolated from peripheral blood mononuclear cells. Upon differentiation, MΦ lose their proliferative abilities and enhance their antibacterial properties, allowing them to participate in the inflammatory and immune responses (Takashiba et al., 1999). By using a scratch assay, we showed for the first time that Phytohustil^®^, and its active ingredient, REAo, concentration-dependently activate the migration of MΦ. Thus, these properties may be associated with an intramucosal chemoattractant activity of Phytohustil^®^ and, the REAo to induce migration of monocyte/MΦ into the injured and inflamed mucosa and may have special effects on the resolution of the inflammation and wound healing (Takashiba et al., 1999; Chanput et al., 2010). These results are suggested as evidence for a positive repair effect against mucosal injury. Chemotaxis of phagocytes to inflammatory site following a release of several cytokine and chemokine is the first step that is decisive for the activation of the host defense (Hsu et al., 2003). Pectic polysaccharides were shown to exhibit potent dose-dependent complement fixating activities, and to induce chemotaxis of MΦ, T-lymphocytes and natural killer cells (Inngjerdingen et al., 2005), a similar mechanism that may explain our results related to the migratory activation of MΦ after treatment with Phytohustil^®^ or the REAo. However, to confirm our *in vitro* data, additional *in vivo* experiments are necessary in the future. Finally, it would be highly interesting to investigate, whether Phytohustil^®^ or the REAo do also reveal anti-inflammatory and anti-oxidative effects on epidermal cells (keratinocytes) *in vitro.*

## Conclusion

The present *in vitro* investigations show a significant anti-oxidant and anti-inflammatory activity of Phytohustil^®^ or REAo- an active component of Phytohustil^®^ – in MΦ, with additional effects on cellular integrity and migratory capacity. The anti-inflammatory effects of Phytohustil^®^ or REAo were similar or even better than effects of diclofenac. These findings may support the therapeutical effects of Phytohustil^®^ observed in patients during the treatment of irritated mucosal membranes and appropriate for symptomatic treatment of dry cough.

## Data Availability Statement

All datasets generated for this study are included in the article/Supplementary Material.

## Author Contributions

GB and RK were responsible for the conception and design of the study. KB, PH, and HS for the data collection, analysis, and image processing. E-UH, performed the HPLC. GB drafted the manuscript and RK revised it critically for important intellectual content. JM, CF, and HA-K discussed the study concept and were responsible for the final approval of the version to be submitted. All authors read and approved the final manuscript.

## Conflict of Interest

CF, HA-K, and JM are employed by Steigerwald Arzneimittelwerk GmbH. We confirm that we have the correct permissions from the copyright holder to publish a research article about this product. The remaining authors declare that the research was conducted in the absence of any commercial or financial relationships that could be construed as a potential conflict of interest.
