# Supplementary figures and images for: Anti-inflammatory and Anti-oxidative Effects of Phytohustil® and Root Extract of Althaea officinalis L. on Macrophages in vitro
Source: Front Pharmacol. 2020 Mar 17;11:290. doi: 10.3389/fphar.2020.00290 (PMC7090173; doi:10.3389/fphar.2020.00290)

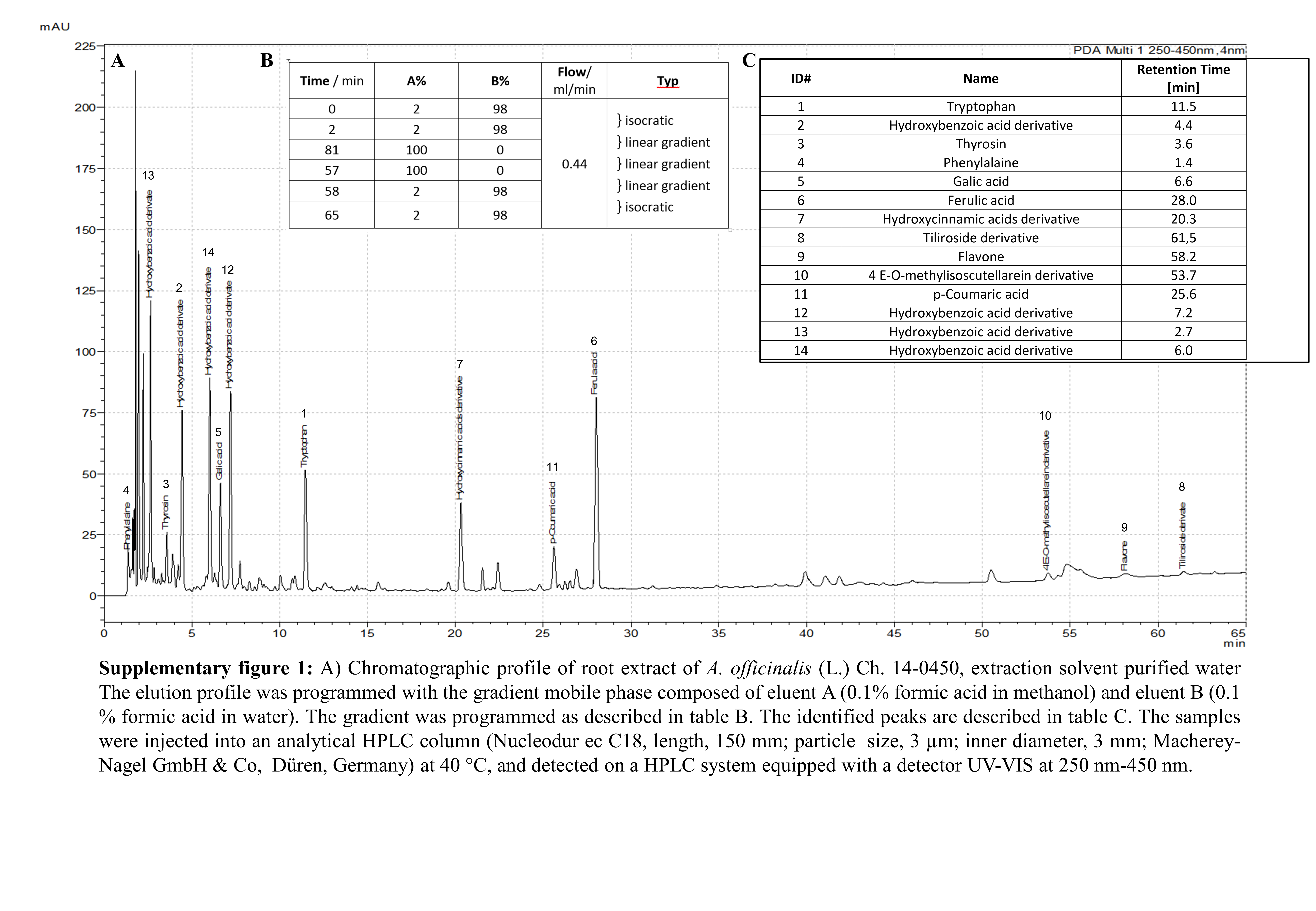

Supplement: Supplementary file 1 [file Image_1.TIF]
